# Supplementary material for: MALDI-TOF MS Approaches for the Identification of the Susceptibility of Extended-Spectrum β-Lactamases in Escherichia coli
Source: Microorganisms. 2023 May 9;11(5):1250. doi: 10.3390/microorganisms11051250 (PMC10224537; doi:10.3390/microorganisms11051250)
Supplement: Supplementary file 1 [file microorganisms-11-01250-s001.zip › microorganisms-2344733-supplementary.pdf]

(μg/mL)

	1	2	3	4	5	6	7	8	9	10	11	12
A	ABPC 32	16	8	2	1	0.5	FMOX 32	16	8	S/C 32/32	16/16	8/8
B	PIPC 64	32	16	8	4	2	AZT 16	8	4	MINO 8	4	2
C	CTX 32	16	8	4	2	1	FOM 128	64	32	MEPM 16	8	4
D	CTX/CVA 4/4	2/4	1/4	0.5/4	0.25/4	0.12/4	AMK 32	16	8	GM 8	4	2
E	CAZ 32	16	8	4	2	1	LVFX 8	4	2	CPFX 4	2	1
F	CAZ/CVA 4/4	2/4	1/4	0.5/4	0.25/4	0.12/4	CAZ 64	CAZ※ 16	8	4	2	1
G	CPDX 32	16	8	4	2	1	IPM 32	IPM※ 8	4	2	1	0.5
H	CPDX/CVA 4/4	2/4	1/4	0.5/4	0.25/4	0.12/4	IPM 16	8	4	2	1	Control

CAZ※(F8-12) and IPM※ (G8-12): including dipicolinic acid (DPA).

Figure S1. The layout of antibiotics and their concentrations on the DPD-1 plate.
